# In Vitro Assessment of the Neuro-Compatibility of Fe-20Mn as a Potential Bioresorbable Material for Craniofacial Surgery

**DOI:** 10.3390/medicina60030440

**Published:** 2024-03-07

**Authors:** Sara Ajami, Charlotte Kraaneveld, Maarten Koudstaal, David Dunaway, Noor Ul Owase Jeelani, Silvia Schievano, Chiara Bregoli, Jacopo Fiocchi, Carlo Alberto Biffi, Ausonio Tuissi, Alessandro Borghi

**Affiliations:** 1 UCL Great Ormond Street Institute of Child Health, London WC1N 1EH, UK; sara.ajami@ucl.ac.uk (S.A.); david.dunaway@gosh.nhs.uk (D.D.); owase.jeelani@gosh.nhs.uk (N.U.O.J.); s.schievano@ucl.ac.uk (S.S.); 2 Erasmus Medical College, Erasmus University Rotterdam, 3015 CN Rotterdam, The Netherlands; 469114ck@student.eur.nl; 3 Oral Maxillo-Facial Surgery, Erasmus MC, 3015 CN Rotterdam, The Netherlands; mjkoudstaal@hotmail.com; 4 National Research Council, Institute of Condensed Matter Chemistry and Technology for Energy, CNR ICMATE Unit of Lecco, 23900 Lecco, Italy; jacopo.fiocchi@cnr.it (J.F.); carloalberto.biffi@cnr.it (C.A.B.); ausonio.tuissi@cnr.it (A.T.); 5 Department of Engineering, Durham University, Durham DH1 3LE, UK

**Keywords:** bioresorbable metals, Fe-20Mn alloys, biocompatibility, cytotoxicity, cell viability

## Abstract

*Background and Objectives:* Spring-assisted surgery is a popular option for the treatment of non-syndromic craniosynostosis. The main drawback of this procedure is the need for a second surgery for spring removal, which could be avoided if a distractor material could be metabolised over time. Iron–Manganese alloys (FeMn) have a good trade-off between degradation rate and strength; however, their biocompatibility is still debated. *Materials and Methods:* In this study, the neuro-compatibility of Fe-20Mn (wt.%) was assessed using standard assays. PC-12 cells were exposed to Fe-20Mn (wt.%) and stainless steel via indirect contact. To examine the cytotoxicity, a Cell Tox Green assay was carried out after 1, 2, and 3 days of incubation. Following differentiation, a neurite morphological examination after 1 and 7 days of incubation time was carried out. The degradation response in modified Hank’s solution at 1, 3, and 7 days was investigated, too. *Results:* The cytotoxicity assay showed a higher toxicity of Fe-20Mn than stainless steel at earlier time points; however, at the latest time point, no differences were found. Neurite morphology was similar for cells exposed to Fe-20Mn and stainless steel. *Conclusions:* In conclusion, the Fe-20Mn alloy shows promising neuro-compatibility. Future studies will focus on in vivo studies to confirm the cellular response to Fe-20Mn.

## 1. Introduction

Craniosynostosis (CS) is the premature fusion of one or more of the cranial sutures, which affects 7.2 of every 10,000 births [1] and causes an abnormal growth of the skull. The shape of the skull depends on the affected suture: the growth is restricted in a direction perpendicular to the fused suture, thereby forcing expansion in the opposite direction. This can cause brain growth restrictions, which could lead to neurological, acoustic, and ophthalmological complications, up to real emergencies [2,3,4,5]. CS may occur in isolation (85%) or as part of a syndromic disease (15%) [6]. Male children are more frequently affected with CS than females (3.5:1) [7].

In a vast majority of cases, the treatment of CS is surgical. Several surgical techniques have been developed to correct the skull dysmorphism associated with CS, such as a cranial vault reconstruction or a modified pi-plasty procedure [8,9]. A minimally invasive option to treat CS is spring-assisted surgery (SAS), which has become an increasingly popular method to treat CS, in both syndromic and non-syndromic cases [10,11]. The surgery entails a small incision over the head to free the fused suture and a temporary placement of spring-like metallic distractors to gradually remodel the skull [12]. The springs used in practice are made of surgical stainless steel. The advantages of this method include smaller skin incisions and less extensive osteotomies, as well as a reduction in the number of blood transfusions, a shorter length of stay in the hospital, and a more rapid postoperative recovery [9,12,13]. However, a major disadvantage of this method is that the springs must be removed after approximately 3 months through a secondary surgery [12]. For this reason, it would be beneficial to replace the stainless steel with a bioresorbable alloy with comparable strength [14]. This would allow for implanting a spring, which exerts the needed force as long as required and then degrades over time, thus removing the need for a second operation.

Bioresorbable metals are generally based on three main alloying systems: magnesium (Mg), iron (Fe), and zinc (Zn) [15,16,17]. Among the three systems, Fe-based alloys have superior mechanical resistance and higher stiffness compared to Mg and Zn, which make them ideal candidates to produce resorbable metallic distractors. Moreover, they exhibit no local or systemic toxicity in both short- and long-term in vivo studies [15,16,17,18,19]. However, their extremely slow degradation rate is the largest barrier for success in clinical application [17], as chronic foreign body reactions will be provoked since Fe would be present in the body for a long time [20]. Pure Fe is also ferromagnetic, which can impede magnetic resonance imaging (MRI). Alloying with sufficient manganese (Mn) promotes the formation of an austenitic phase, improving MRI compatibility and formability [20]. Furthermore, faster degradation rates may be attained through the addition of Mn: rates as high as 1.26–1.3 mm/year have been reported in in vitro studies for Fe-35Mn and Fe-20Mn wt.% [21,22].

Even though Mn is a micronutrient and is biocompatible to a certain degree, it could still have serious side effects [23]: in high concentrations, Mn could have a neurotoxic effect, which, sometimes, even manifests itself as a Parkinson’s disease-like syndrome [24]. The Fe-Mn alloys with a manganese content of 20–35% demonstrated a low inhibitory or no negative effect on osteoblast, fibroblast, or endothelial cell types in a test of cytocompatibility and cell viability [25,26,27]. Moreover, 20–30 wt.% Fe-Mn alloys have similar mechanical properties to stainless steel [21,26]. However, limited information is available on the neuro-compatibility of this material. For applications of bioresorbable alloys in craniofacial surgery, specifically SAS, it is particularly important to assess the neuro-compatibility of Fe-20Mn due to the surrounding neuronal tissues of the implantation site.

Therefore, the aim of this study was to assess the neuro-compatibility of the bioresorbable alloy Fe-20Mn wt.%, with the hypothesis that it induces a similar reaction to a standard surgical-grade alloy—such as stainless steel—when exposed to a suitable cell line. The degradation behaviour of the alloy in pseudo-physiological condition was assessed, and multiple assays were performed on PC-12 cells to examine the in vitro biocompatibility of this material.

## 2. Materials and Methods

### 2.1. Specimen Preparation

Two different materials (Fe-20Mn and AISI316L stainless steel) were used in the present study. Medical-grade AISI316L was acquired as a commercial sheet (Metal Supermarket, London, UK); Fe-20Mn alloy was produced by vacuum induction melting of pure raw materials and cast in a water-cooled copper crucible. The obtained ingot was then annealed at 1100 °C for 3 h under a flowing Ar atmosphere, hot-rolled at 800 °C, cold-rolled into 1.5 mm thick sheets (25% thickness reduction), and finally subjected to annealing at 600 °C for 1 h, followed by water quenching. Disc-shaped (diameter of 11.0 mm and thickness of 1.5 mm) and rectangular (10 × 20 × 1.5 mm) specimens were fabricated by electrical discharge machining. All the specimens were finely polished using silicon carbide abrasive paper (grades #800, and #1200) followed by 6 µm and 1 µm cloths, and finally washed in ethanol with ultra-sonication.

### 2.2. Static Degradation Tests

Static immersion degradation tests were carried out according to the ASTM G31 standard [28] in a modified Hank’s solution for durations matching the extract time points used for cell culture tests (24 and 72 h), and for a further conventional point at 7 days. Rectangular samples were sterilised by immersion into 70% ethanol and suspended by a sterilised nylon wire in the middle of Pyrex bottles containing 100 mL of degradation medium; bottles were finally stored in an incubator under cell culture conditions (5% CO_2_, 37 °C) for the chosen time frames. At the end of the test, the samples were extracted from the degradation medium, ultrasonically cleaned in ethanol, and weighted: the degradation rate (*DR*) was computed according to Equation (1):

(1) $$DR=\frac{8.76\cdot 10^{4}W}{At\rho }$$

where *t* is the time of exposure to the solution (h), *A* is the area of the sample (cm^2^), *W* is the mass loss (g), and *ρ* is density (g/cm^3^). Moreover, detached corrosion products, as well as the surfaces of degraded samples, were characterised by SEM-EDS (Zeiss mod. Leo, Zeiss, Oberkichen, Germany operating at 20 KV) and XRD (Panalytical X’Pert Pro using Cu Kα radiation operating at 40 kV and 30 mA on the xy surfaces of the samples in the 2θ range of 20–120°), so as to understand their nature and composition.

### 2.3. Extract Media

Extraction media were prepared according to ISO 10993-5 [29]. Prepared samples were sterilised by immersing into 70% ethanol (3×—15 min washes) and by subsequent UV exposition (3 h). The Fe-20Mn and stainless steel discs were incubated in RPMI 1640 media (Gibco, Fisher scientific, Leicestershire, UK) supplemented with 0.5% fetal bovine serum (FBS) (GIBCO, Fisher scientific, Leicestershire, UK) and 1% horse serum (HS, HI, GIBCO, Fisher scientific, Leicestershire, UK) with surface area or mass/volume of 3 cm^2^/mL in sterile 15 mL tubes under cell culture conditions (5% CO_2_, 37 °C). In order to illustrate the influence of extract time, two extract time points (24 and 72 h) were set with the same extract environment. Six samples were used for each material per time point. After the extract time points, the discs were taken out of the media. The media of the same extract time point were mixed to ensure a homogenous extract medium.

### 2.4. Cell Culture Plate Coating

The surface of the well plates was modified with collagen IV to allow for the adhesion of PC12 cells. Then, 0.115 mL Acetic Acid (Glacial acetic acid, reagentPlus, 99%, Sigma Aldrich, International, St. Luis, MO, USA) was dissolved in 20 mL deionized water. In addition, 2 mg of Collagen IV (Sigma Aldrich, International, St. Luis, MO, USA) was reconstituted for 3 h while stirring at room temperature. The solution was then transferred to an autoclaved screw-capped glass bottle, and an aliquot of chloroform (Sigma Aldrich, International, St. Luis, MO, USA—10% volume collagen solution) was added to sterilise the solution. After overnight incubation at 4 °C, the top-layer collagen was aseptically removed and stored in sterile tubes at 4 °C. Then, the surface of the well plates was coated with a sufficient amount of collagen to cover the surface and left overnight in a 37 °C incubator; excess fluid was removed, and well plates were sterilised through exposure to UV light for 3 h in a sterile culture hood followed by rinsing with 70% ethanol.

### 2.5. Cell Culture

The PC12-cell lines from rat adrenal gland (phaeochromocytoma) were derived from Sigma Aldrich (88022401-1VL) and were maintained in RPMI 1640 + 2 mM Glutamine + 10% HI + 5% FBS (complete culture media). After reaching 70–80% confluence, PC-12 cell suspensions were sub-cultured and seeded at a density of 5000 cells per well in 96-well plates coated with collagen IV (Sigma Aldrich, International, St. Luis, MO, USA) in complete culture media for 24 h to allow for attachment. Then, the media were replaced, and cells were introduced into a differentiation medium consisting of an RPMI-1640 medium containing 0.5% FBS, 1% HS, 2 mM L-glutamine, and 100 ng/mL nerve growth factor β (NGF-β, Sigma-Aldrich, International, St. Luis, MO, USA). Cells were exposed to this differentiation medium for 6 days to be able to differentiate into neuron-like cells. A differentiation time of 6 days was chosen because of studies on PC-12 differentiation and neurite outgrowth which showed that neurite outgrowth started to plateau on day 6 [30].

Subsequently, the cells were treated per 5 replicates with the Fe-20Mn and stainless-steel extract media. The negative control consisted of cells cultured in differentiation media only. Then, the media were replaced every two days, and the cells were maintained in the differentiation media for 6 days.

### 2.6. Cytotoxicity

To assess cytotoxicity, PC12 cells were treated with 100 µL of the extract media for 24, 48, and 72 h, and a CellTox Green Cytotoxicity Assay (Promega, Madison, WI, USA) was performed. This assay detects changes in the membrane integrity of cells as a result of cell death. The CellTox green dye is nontoxic and only binds the DNA in compromised cells. As a result of this, the fluorescent properties of the dye are enhanced, and the fluorescent signal produced is proportional to cytotoxicity. At the end of the first time point, 100 μL of the CellTox Green reagent (2×) was added to each well. A lysis solution was used as a positive control. The lysis solution was added to the cells before reading the results after cells had been incubated with normal cell culture media only.

The same plate was read continuously at all time points. Plates were incubated for 15 min at room temperature, shielded from light, before measuring the results. Fluorescence intensity was measured using a Spectramax i3X microplate reader (Molecular Devices, San Jose, CA, USA) at an excitation wavelength of 485 nm and emission of 535 nm. Wells with only cell culture medium were used as a control for the readings. The measurements were then standardised against these control readings.

### 2.7. Morphological Examination

Neurite outgrowth is an important morphological indication of healthy cell functioning in PC-12 cells [31]. A total of 4000 cells/well were seeded in collagen (IV)-coated 12-well plates containing a 1 mL/well complete medium. The cells were incubated with the differentiation medium for 6 days, and at the end of the differentiation time, they were treated with the extract medium for 1, 3, and 7 days. The cells were fixed with 4% paraformaldehyde (BioLegend, San Diego, CA, USA) for 20 min and washed three times with PBS. This was followed by permeabilization with a 0.1% triton X-100 (Sigma Aldrich, St. Luis, MO, USA) solution in PBS for 10 min. The cells were then blocked with 5% normal goat serum (Sigma Aldrich, USA) in PBS to block nonspecific binding for 60 min, followed by incubation with primary antibody Recombinant Anti-beta III Tubulin antibody [EP1569Y] (Abcam, Cambridge, UK) (0.095 mg/mL) diluted in 1% goat serum overnight at 4 °C. Conjugated secondary antibodies (Goat anti-rabbit IgG H&L, Abcam, Cambridge, UK) were applied for 2 h at room temperature, protected from light, and the nuclei were counterstained by Hoechst (Thermo Scientific, Karlsruhe, Germany).

Images were acquired using an Olympus IX71 microscope with a magnification of 20×. This was chosen at the time of imaging to have approximately 10–20 cells in view per image. A total of 60 cells per extract medium type were analysed. The images were analysed for the number and length of the neurites and the cell body area. To measure the neurite length, the longest possible route per neurite was measured if a neurite had multiple branch points. For an overview of the well, images with 10× magnification were also captured.

The analysis of the images was performed using ImageJ (Fiji v.1.53o) software (https://imagej.nih.gov/ij/, accessed on 1 February 2022). Using the freeline tracing tool in ImageJ, neurites and cell bodies were measured manually. Cells were excluded from the analysis when they were on the edge of the image and part of the cell was not in the field of view. Cells were also excluded if they were clumped together and no distinction could be made between the cells or their neurites. Results of these measurements were imported into Excel for further statistical analysis.

### 2.8. Data Analysis

A statistical analysis was performed with all obtained data from the experiments. SPSS IBM was used to run the statistical analysis. To determine if the data had a normal distribution, a test of normality was performed (Shapiro–Wilk). The results of the cell toxicity test showed that the data did not have a normal distribution. Therefore, the median ± the interquartile range is reported. A Kruskal–Wallis test was performed to find differences. If a significant difference was found, the Kruskal–Wallis was followed by a non-parametric, 2-independent-samples test.

The results of the morphological examination showed a normal Gaussian distribution; the mean ± the standard error of the mean of all experiments was determined followed by an unpaired *t*-test or an ANOVA analysis. A Tukey post hoc test was performed to find differences in the data after the ANOVA analysis. In this study, a *p*-value of 0.05 or lower was considered significant.

## 3. Results

### 3.1. Static Degradation Behaviour

**Figure 1:**
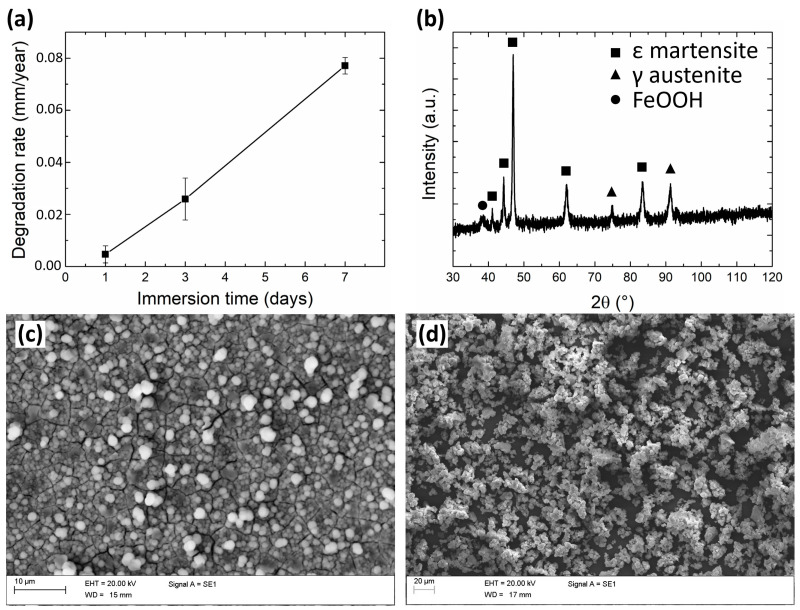
(**a**) graph depicting the evolution of degradation rate during static immersion tests; (**b**) XRD pattern and (**c**) SEM image of the surface of an Fe-20Mn sample after 7 days of immersion; (**d**) SEM image of corrosion products collected from the bottom of bottles after 7 days-long degradation tests.

The degradation rate of the Fe-20Mn alloy (Figure 1a) was found to increase almost linearly with the incubation time: in particular, it increased from 0.005 mm/year after 1 day to 0.026 mm/year after 3 days and 0.077 mm/year at 7 days of immersion. After immersion for 7 days, the surface of the degraded samples appeared to be partially covered by a scale of degradation products, while some areas displayed a bare metallic surface owing to the partial detachment of the scale itself. According to XRD and EDX analyses, such degradation products consisted of a base layer of iron hydroxide FeOOH (Figure 1b) whose surface is decorated by (Fe,Mn)CO_3_ scattered cuboids [32,33]. In particular, the FeOOH layer assumed its crystalline α goethite form [34] (Figure 1c). The degradation products detaching from the metallic surfaces during incubation (Figure 1d) were found to be rich in O, Na, Ca, and P, as well as Fe and Mn: they likely consisted of hydrated iron oxides, together with reduced amounts of phosphates [35].

### 3.2. Cytotoxicity

**Figure 2:**
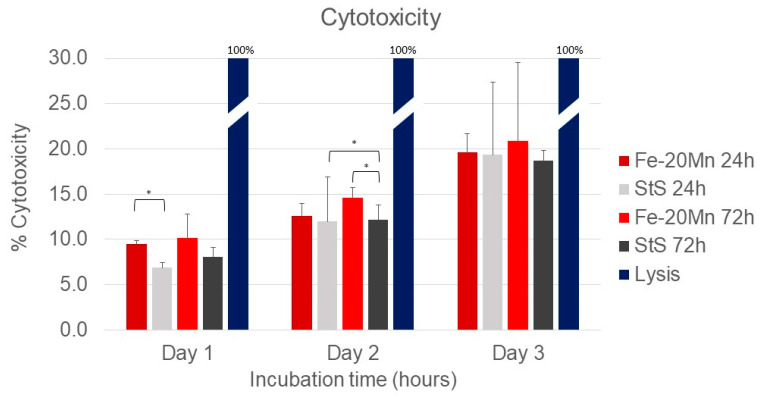
Cytotox green assay results using PC 12 cells after 24, 48, and 72 h. Results were measured in relative fluorescence units. The lysis solution was used as a toxic control and set as a 100% toxic compound. The other extract media types were normalised against this positive control. * Significant differences *p* < 0.05.

Figure 2 presents the results of the cytotoxicity assay. All types of extract media scored significantly lower than the lysis solution (*p* < 0.001).

On day 1, the percentage cytotoxicity of the 24 h Fe-20Mn (9.5% [9.1–9.8]) extract medium was significantly higher than the extract medium of the 24 h stainless steel (7.3% [6.8–7.4]) (*p* = 0.009). At later time points, the difference between the two 24 h extract media types was no longer present. After 2 days of incubation time, the 72 h Fe-20Mn extract medium (14.1% [13.6–15.8]) was significantly more toxic than the stainless-steel 72 h (12.1% [10.6–13.8]) (*p* = 0.047). The 24 h stainless steel (11.7% [11.5–12.5]) was significantly more toxic than the 72 h stainless steel, too (12.1% [10.6–13.8]) (*p* = 0.047).

No differences in cell toxicity between the four types of extract media were found after 72 h of incubation. When the days were compared with each other, all extract medium types became more toxic over time.

### 3.3. Morphological Examination

**Figure 3:**
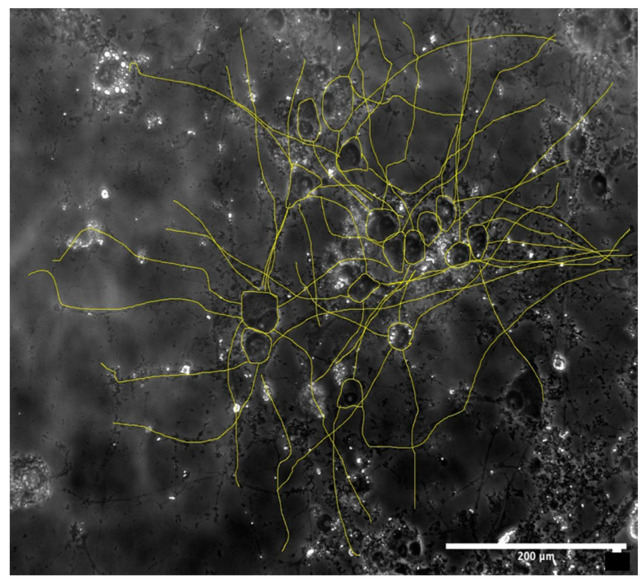
Example of image analysis of a 72 h Fe-20Mn brightfield image on day 7. Scale: 200 μm.

After the cell toxicity assays, the morphological examination was carried out. Figure 3 shows a sample of the analysis of the morphological examination. All microscope images were captured at a 20× magnification to capture approximately 10–20 cells per image. Cells were not included if they were on the edge of the field of view or if their neurites were out of the field of view.

**Figure 4:**
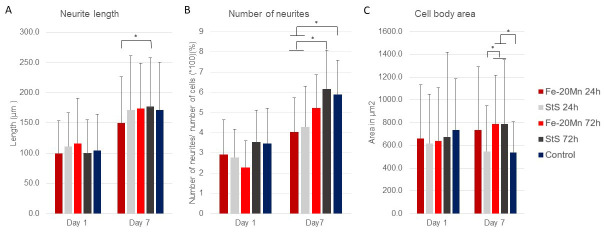
(**A**) Average neurite length with standard deviation after 1 and 7 days of incubation time to extract media. (**B**) Number of neurites per cell with standard deviation when exposed to different types of extract media. The number of neurites was standardised by dividing the average by the total number of cells analysed (60 each group) × 100. (**C**) Average cell body area with standard deviation of PC12 cells exposed to different types of extract media after 1 and 7 days of incubation. * Significant difference.

In Figure 4A, the results of the image analysis are presented. Interestingly, after 1 day of incubation time, cells that were given the 72 h Fe-20Mn extract medium had the longest neurites (116.1 ± 8.2 μm). However, no differences in average neurite length were found between the different types of extract media or the control on day 1. After 7 days of incubation time, the neurite length of the cells increased in all groups. Cells that were given the 24 h Fe-20Mn extract medium had the shortest average neurite length (149.7 ± 6.3 μm), and cells that were fed with the 72 h stainless steel extract medium had the longest average neurite length (176.9 ± 6.0 μm). The difference between these two types of extract media was the only significant difference found within the day 7 time point (*p* = 0.19, CI −51.4; −2.9).

Figure 4B shows the results of the number of neurites per cell. No differences in the percentage of number of neurites were found on day 1. After 7 days of incubation time, the number of neurites per cell increased in all groups, and there were differences between the types of extract media.

The 72 h stainless steel (6.17 ± 1.9%) had a significantly higher number of neurites per cell than the 24 h Fe-20Mn (4.03 ± 1.7%) (*p* = 0.002, CI 0.35; 2.25) and 24 h stainless steel (4.28 ± 2.0%) (*p* = 0.009, CI 0.20; 2.11). Similarly, the control (5.89 ± 1.7%) had a higher number of neurites per cell than the 24 h Fe-20Mn (4.03% ± 1.7) (*p* = 0.005, CI 0.24; 2.05) and the 24 h stainless steel (4.28 ± 2.0%) (*p* = 0.022, CI 0.09; 1.91).

Lastly, the cell body area was analysed. After 1 day of incubation, no differences in cell body area were found between the cells that had been exposed to different types of extract media (Figure 4C). On day 7, however, a difference was found in cell body area. Cells that were exposed to 72 h StS were found to have a significantly smaller cell body area than cells that were exposed to the 72-Fe-20Mn extract medium (*p* = 0.029, CI −469.5; −15.9) or the 72-Sts extract medium (*p*= 0.22, CI −477.3; −23.6). Similarly, the 72 h Fe-20Mn (*p* = 0.022, CI 23.6; 477.3) and 72 h stainless steel (*p* = 0.037, CI 9.6; 491.6) extract media had larger cell bodies than the control.

**Figure 5:**
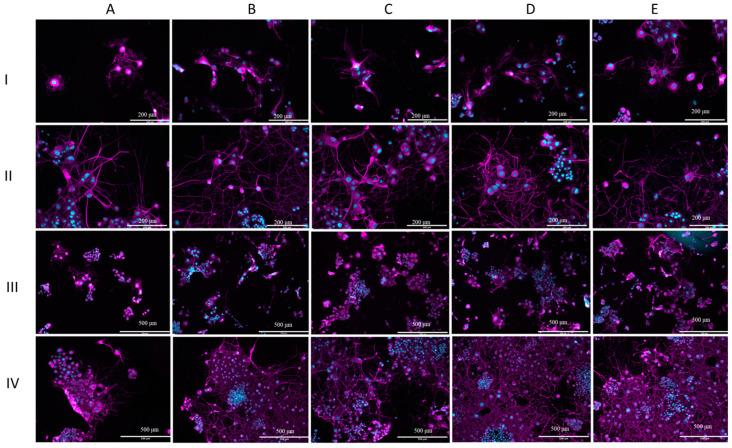
Morphological assessment. (**I**) Fluorescent microscope images (20×) showing cell morphology of PC12 cells after 1 day of incubation. (**II**) Fluorescent microscope images (20×) showing cell morphology of PC12 cells after 7 days of incubation. (**III**) Fluorescent microscope images (10×) showing cell morphology of PC-12 cells after 1 day of incubation. (**IV**) Fluorescent microscope images (10×) showing cell morphology of PC-12 cells after 7 days of incubation. On each line: (**A**) cells exposed to 24 h Fe-20Mn extract medium, (**B**) cells exposed to 24 h StS extract medium, (**C**) cells exposed to 72 h Fe-20Mn extract medium, (**D**) cells exposed to 72 h StS extract medium, (**E**) control. Cell nuclei were stained with Hoechst and display blue here. Recombinant Anti-beta III Tubulin antibody was used to stain tubulin. Tubulin is depicted as magenta in the images.

Figure 5 displays representative images captured with the fluorescence microscope. When comparing days 1 and 7 (Figure 5I,II), it can be observed that cells exposed to all types of extract media neurites grew extensively in terms of length and number. On day 1, neurite outgrowth was observed in all groups, but not all cells had neurites. Similar to the results of the analysis, there was little apparent difference between the groups on day 1. The difference in neurite number between the 24 h Fe-20Mn and 72 h stainless steel and the control can be noticed in Figure 5(AI,DI). The neurites of the 72 h stainless steel covered greater distances than the neurites of cells exposed to the 24 h Fe-20Mn. On day 7, the neurites can be seen to overlap and even make connections with other neurites (Figure 5(BII)).

Lower-magnification (10×) images are presented in Figure 5III,IV. Similar to the higher-magnification images, the increase in neurite growth, neurite number, and cell growth between days 1 and 7 can be observed. Large clumps of small cells can clearly be seen at the lower magnification, especially on day 7 (Figure 5IV).

On day 7, it can also be observed on this magnification that, especially for 72 StS and the control, the field of view is almost completely covered with cells and neurites. Cells that were exposed to the 24-h Fe-20Mn extract medium seemed to remain behind in this process of growth (Figure 5(AIII)).

The results of the analysis of the cell body area showed a smaller average cell body area for the 24 h stainless steel and the control. Looking at Figure 5(BIV), it can be noticed in the lower magnification that cells overlap, but a difference in cell body area is not noticeable by observation only.

## 4. Discussion

The present work aimed to assess the neuro-compatibility of the bioresorbable Fe-20Mn wt.% alloy. This alloy was compared to medical grade stainless steel and assessed in terms of cytotoxicity, cell viability, and cell morphology. This study, which, for the first, time examined the biocompatibility of an FeMn alloy using a neuron-like cell line, could be useful for further research into its biocompatibility.

### 4.1. Static Degradation Behaviour

The degradation behaviour of the alloy was evaluated by immersion under static conditions, yielding the results reported in Figure 1. A linear behaviour was found for the degradation rate. Such continuous increment, which is consistent with the available literature data [36], is expected to level out for longer times, leading to a relatively stable behaviour owing to the formation of a partially protective scale of degradation products [37]. Although the discussed degradation products can be expected to seize a considerable amount of Fe^2+/3+^ and Mn^2+^ ions produced by the corroding alloy, a certain amount of such ions will remain dissolved in the solution, possibly affecting the surrounding biological environment.

### 4.2. Cytotoxicity

The possible cytotoxic effect of Fe-20Mn has been studied in previous research. Hermawan et al. [26] explored the effect of several FeMn alloys (Fe-20Mn, Fe-25Mn, and Fe-30Mn) on fibroblast cells compared to medical-grade stainless steel and reported no cytotoxic effects. Similarly, Dargush et al. [25] and Chou et al. [38] both carried out cytotoxicity assays after exposure to FeMn extract media but used pre-osteoblast cell lines. No toxic effects were found by Dargusch et al. [25] after 1 and 3 days. Chou et al. [38] found a high cell viability after 3 days but an initial inhibition on day 1. Only Schinhammer et al. [38] reported a slightly reduced, but still acceptable, cell viability using human umbilical vein endothelial cells.

The results of these previous studies are in line with the ones reported herein, as after 3 days, there was no difference in toxicity between Fe-20Mn and stainless steel. However, this study demonstrated that at earlier time points, Fe-20Mn was more toxic than stainless steel, similar to the results of Chou et al. [38].

In all types of extract media, the cytotoxicity became higher over time, which may be due to the used protocol: due to the nature of the Celltox Green assay, it was not possible to change the media of the cells. Therefore, although cell death may also be due to a lack of nutrients, it is safe to assume that all groups were equally affected. A future study may confirm the present results by using another type of assay, such as the MTT assay which was employed in several other studies [25,26,38,39].

The International ISO 10993-5 [29] guideline mandates the measurement of cytotoxicity in order to determine if a compound is suitable for use in medical devices. This standard provides two options to test the biocompatibility: direct or indirect contact of the material and the cells.

In the present study, indirect testing was employed, whereby only the degradation product of the discs is exposed to the cells. Previous research by Scarcello et al. [40] regarding the degradation of Fe hypothesized that the process of degradation of Fe—whereby hydroxyl radicals (OH^−^) form—is the main cause of cytotoxicity: indirect assays may, therefore, underestimate possible OH^-^ toxicity. Whether this process is also dangerous with Fe-20Mn degradation is not included in this study. Although direct contact was not included in this study, two previous studies have tried to examine the cell response after direct contact with Fe-20Mn with varying results. Chou et al. [38] exposed pre-osteoblast cells directly to FeMn scaffolds. In the study, cell viability was measured using a live/dead assay after 1 and 3 days. After 3 days, the scaffold was covered in living cells, suggesting a good compatibility [38]. Andreas Drynda et al. [41] also performed a direct exposure test and found good cell viability using a live/dead assay after 1 and 3 days. Cells were growing on top and near the discs. However, after 6 and 10 days, the inhibition of cell growth was observed around the discs, and cells were no longer growing on top of the discs. These results could mean that FeMn is not toxic in lower concentrations, but it does have an inhibiting effect in higher concentrations or longer exposures. Nevertheless, the researchers commented that the highest concentrations after 6 and 10 days were a consequence of the design of the experiment and were unlikely to appear in vivo in a dynamic environment [41].

### 4.3. Morphological Examination

In assessing the neuro-compatibility of a potential toxic compound, not only cytotoxicity is useful, but assessing cell morphology can provide additional and important information. The in vitro examination of cell morphology provides an opportunity to study neuronal development at a cellular level [42]. Neurite outgrowth is a crucial process in the development of neural tissue, and a disruption can lead to various cognitive disorders, for example mental retardation [43,44]. PC-12 cells have been used to examine the potential toxic effect of multiple chemicals. Neurite length and the number of neurites give an indication of the health of PC-12 cells [45]. Even tough in vitro studies cannot be directly related to the in vivo situation, examining the morphology of the cells can give an indication of potential toxicity in vivo [45].

This is the first study to comprehensively assess the effect of Fe-20Mn on the cell morphology of neuron-like cells. Neither neurite length nor the number of neurites or cell bodies differed on day 1 in the analysis of cell morphology; thus, it can be assumed that the cells were not affected by the Fe-20Mn extract media. Although the results of day 7 varied, both the neurite length and neurite number appeared to be affected by the 24 h Fe-20Mn extract media. This could also be observed in the lower-magnification images. Interestingly, the cells that were fed with the 72 h extract media were not affected and grew equally as good as the control. This is remarkable considering that 72 h Fe-20Mn was expected to have degraded more and, thus, have a more toxic effect.

Dargush et al. [25] performed a morphological examination after pre-osteoblast cells had been exposed to Fe-20Mn extract media for 1 and 3 days. The cells were not affected by the Fe-20Mn and displayed the same morphological features as the control. These results are in line with our study because, in this study, no morphological differences were also found on day 1. However, Dargush et al. only carried out the morphological examination after 1 and 3 days, whereas, in this study, the cells only seemed to be affected after a longer period of exposure. Moreover, the results of their study were based only on observations, and images were not analysed systematically. No other studies were found to have reported a morphological examination after exposure to Fe-20Mn.

### 4.4. Limitations

Although this work employed a well-established protocol to assess the biocompatibility of Fe-20Mn, a few limitations should be mentioned.

The results from the degradation tests showed a very consistent behaviour, and the degradation speed of the material appeared to increase linearly over time. However, the laboratory preparation of the extraction medium showed less consistency as different dissolution tubes examined at the same timepoint had different amounts of degradation products: to minimise inconsistency, the extract media from the different tubes from the same time points were mixed with one another to form a homogeneous extract medium. Although this degradation process was not quantified (tests were carried out in two separate institutions), cytotoxicity tests showed good repeatability and a sensible trend. However, larger clusters of Fe-20Mn degradation products could be observed in some of the wells (such as the one in Figure 3, where they are visible as small black marks).

The analysis of neurite morphology was carried out using a well-known image processing software (ImageJ Fiji v.1.53o). Manual segmentation was performed, and, therefore, a certain amount of operator-dependence could be expected; furthermore, distinguishing single-cell morphology proved complex in the presence of overlapping neurites and their cell bodies. The analysis method employed in this study is consistent with previous studies, and future works will employ automated methods [46] to extract neurite morphology.

## 5. Conclusions

The results of this work seem to confirm the initial hypothesis that the performance of Fe-20Mn in terms of neuro-compatibility is similar to that of stainless steel, both in terms of cytotoxicity (after day 3, Figure 2) and in terms of the qualitative (Figure 5) and quantitative (Figure 4) morphological assessments of neurite outgrowth at different incubation times and concentrations.

These results are a first step in the in vitro assessment of Fe-20Mn as a bioresorbable, neuro-compatible material and show that the alloy could potentially be a biocompatible material. Initial tests on early cell viability (not reported here) confirmed the results of the cytotoxicity assays. A thorough assessment will be carried out in the future to confirm the present findings.

In vitro assays alone cannot determine if a material is biocompatible because cells behave differently when surrounded with other cell types, cell signalling factors, and extracellular matrices. Therefore, future studies should focus on an in vitro tissue culture or in vivo animal studies to investigate further whether Fe-20Mn would also be a biocompatible material for future use in craniofacial surgery.

## Data Availability

The data that support the findings of this study are available from the corresponding author, A.B., upon request.
